# Ethical globalization? Decolonizing theoretical perspectives for internationalization in Canadian medical education

**Published:** 2018-05-31

**Authors:** Taqdir Bhandal

**Affiliations:** 1 Social Justice Institute, University of British Columbia, British Columbia, Canada

## Abstract

**Background:**

Internationalization is a process being undertaken at institutions of post-secondary education worldwide in response to globalization. The resulting imperatives for medical education include re-thinking Canadian positionality within uneven, problematic Global North-South relations. Members of the medical education community are in need of training and tools to navigate this complex situation.

**Methods:**

Using a decolonial theoretical perspective, a literature review was conducted and framed with three ethical questions posed to stimulate collective conversations about internationalization among all members of the health professions education community in Canada.

**Results:**

This study identifies analytical gaps in discussions on the role of medical education in the context of colonial, neoliberal, unjust Global North-South relations. The results point to the need for deeper examination of medical curricula for problematic representations and theorization of inequities and racialization. They also suggest that practices for International Medical Electives and the involvement of International Medical Graduates should be evaluated in light of the ethical concerns identified.

**Conclusion:**

During this moment of internationalization and globalization in all health professional education, reflexivity and self-awareness are important strategies for engaging with decolonizing theoretical perspectives that are critical of Global North-South relations like neoliberal globalization and colonialism. Increased inclusion of pluralistic ways of approaching both processes are necessary for combatting growing health inequities in Canada and globally.

## Introduction

In line with many of the emerging debates in post-secondary education, the discourses of “globalization” and “internationalization” have become important contributors in the conversations of medical education practice and policy.^1-7^ In relation to contemporary political economies, globalization is a multi-level process signalled by the rapid advancement in technology, transnational economic trade, and international dispersion of humans, plants, animals, cultures, and worldviews, and has taken on a particular form in the last thirty years.^8-10^ While the movement, mixing, and colonizing of people and cultures has been going on for thousands of years, this paper focuses on the timeframe of “neoliberal globalization” dating back to the late twentieth century. Through the imposition of debt and unequal trading of goods and services, neoliberal globalization began as a social, political, and economic process where the flow of financial and cultural capital among countries of the Global South and Global North increased exponentially. I define the term Global South as encompassing previously colonized countries, which are primarily located in the Southern Hemisphere. While these countries are no longer colonies (under full legal, political, and social control by Euro-American nations such as Britain, France, and Portugal), the impacts of colonial policies and practices continue to influence their political economies and people in powerful ways. The Global South include parts of Asia, Africa, South America, and the Middle East. In global health and international development fields, the term “North-South Divide” is used to denote the differences in politics, size of economies, and cultures. Northern countries are seen as developed, Western, predominantly white, educated, industrialized, secular, and high income while Southern countries are often classified as developing, Eastern, racialized, having strong ties between religion-government-public life, over populated, low income, impoverished, and in need of aid. From a critical, decolonial perspective, neoliberal globalization has propagated an international economic, racialized, and gendered system that gives more power to Northern countries, perpetuates Euro-American-centric beliefs and worldviews, erases the local knowledges of Southern countries and Indigenous peoples in the Global North and South, and orients land and nature as resources to be exploitatively extracted for human consumption. Many authors have written on the impact of neoliberal globalization on the health of racialized and non-racialized peoples and health systems.^11-13^

At the same time, institutions of post-secondary education across the globe are undergoing the process of internationalization in which they aim to foster ethno-cultural and national diversity in their curriculum, hiring practices, Northern and Southern student recruitment policies, and other components of their strategic plans and programs. In one of the most ethno-culturally diverse countries in the world with unique commitments to the principles of health equity nationally and globally,^14^ members of the Canadian medical education community should be aware of the current debates surrounding globalization and internationalization in post-secondary education and critical social justice literature. Decision-makers and medical educators would benefit from evidence-based, equity-oriented primers for the boardroom to the classroom about what the two processes mean for their institutions, teachers, students, and themselves.

The primary aim of this paper is to provide a reflexivity and self-awareness tool for Canadian health professions education administrators who are thinking about and practicing internationalization in their home institutions. As an identified tool for knowledge translation, I use the exercise of ethical questioning to enable readers to identify, understand, raise self-awareness and relate to^15^ the nuanced challenges of decolonial approaches to globalization and internationalization in medical education. In this paper, I provide a brief overview of the neoliberal globalization literature (which is immense) and subsequently address three important ethical questions (among many) to be asked on the topic of internationalization in the medical education context. The first two questions discuss in detail the implications of both processes for curriculum and International Medical Electives (IMEs). The last question briefly touches on considerations for International Medical Graduates (IMGs) in Canada. This tool can also be useful for teachers and students to think about how these questions might apply pedagogically to curriculum and learning. It can also be used for policy makers who may be in the process of (re)writing internationalization policies. Through the review of relevant literature, I aim to provide a pathway for mediating and addressing past and present Global North-South and Indigenous colonial relationships that are reproduced in policies and practices in medical and other health professional education.

### Thinking through decolonization for medical and other health professional education

Numerous Canadian scholars have written about the importance of having a strong theoretical orientation for doing research on health and wellness.^16,17^ Readers are encouraged to engage with the other articles on globalization in *Canadian Medical Education Journal* as a means of understanding the multiplicity of perspectives that can be taken when thinking about this process. In line with the tenets of global health equity and sustainable development, this paper uses a decolonizing theoretical perspective to make sense of the inter-relation between health equity, Global North-South power relations, commitments to Indigenous peoples’ self-determination and how we can work together to reimagine what ethical relationships can look like. There is a rich history and diversity of standpoints within critical theories of racialization. Five main traditions are Indigenous decolonial theory, intersectionality or Black feminist theory, postcolonialisms, critical race theory, and planetary humanism. For the purposes of this paper I use the active term *decolonizing theoretical perspective* to denote a research and practice perspective which aims to move forward the politics of decolonization, defined as the “building of mass movements capable of dismantling settler-colonialism, white supremacy, and capitalism.”^18^ A decolonizing theoretical perspective suggests that all knowledge is partial, incomplete, and changing; moreover, it posits that there are deep connections between the construction of knowledge and the utilization of earth’s resources such as land and water in our Northern societies and the ongoing marginalization of racialized groups in Canada and the world over. For educators, the epistemology or philosophy of knowing is a central point around which we position learning experiences. The worldview in which we base the mission of the educational institution has implications for curriculum, pedagogical practices, and international, national, and inter-provincial relationships.

The Truth and Reconciliation Commission on Residential Schools, Thomas Dignan’s (Chair, Royal College Aboriginal Health Advisory Committee) statement calling the state of Indigenous health in Canada a national embarrassment, the rise in reporting of racialized discrimination targeting Muslims in Canada,^19^ youth driven social movements like Black Lives Matter Vancouver, Toronto, Halifax, etc., and Canadian Institutes of Health Research priority announcements on Indigenous and refugee health^20-21^ all signal the importance of addressing discourses and material practices that create unjust and avoidable health inequities across racialized lines locally, nationally, and globally. They are also reflective of global mobilities and Canadian commitments to redress the inter-generational and ongoing consequences of epistemological hegemony, or the dominance of Northern philosophies of knowledge over Southern ones.^22-24^ This means attending to our national historical position as a mostly British and French settler-colony and our current position as home to people of very diverse cultural backgrounds and worldviews.^25^ From a pedagogical perspective, this also requires an exploration of epistemic pluralism and how land and borders both structure and are structured by our understandings of health and healing.

In this paper, I aim to undertake a critical analysis of approaches to globalization and internationalization through a decolonizing theoretical perspective in order to disrupt the common sense belief that these two processes are beneficial and equitable for all Northern and Southern participants. I aim to provide information to raise awareness of the importance of decolonial perspectives in post-secondary education internationalization policies. Also, I aim to foster a common language for dialogue between medical education, internationalization policy, and decolonizing theoretical perspectives. I hope that this article provides an important reference for a decolonizing conversation on globalization and encourages the redressing of practices that reproduce inequities.

### The contested definitions of globalization and internationalization

An important distinction to make when writing, thinking, and planning policy and practice is the difference between the meta-narratives of “globalization” and “internationalization.” Globalization has come to mean to the theoretical and material exchange of earth-based resources, ideas, money, and people across the world; internationalization refers to the pointed recognition and response within institutions of increasing ethno-cultural diversity. According to the Organization for Economic Cooperation and Development, globalization is “a dynamic and multidimensional process of economic integration whereby national resources become more and more internationally mobile while national economies become increasingly interdependent.”^26^ As the reader can see from this definition, the current trajectory of globalization is closely tied to a market-driven political economy that spans North-South. For the medical profession and health professional educators, international trade has been shown to influence the global network of health human resources and students, access to primary health care, and access to pharmaceutical distribution around the globe among other transnational transactions.^27,28^ From a social determinants of health perspective, such transactions represent structural, macroscopic processes that strongly influence our collective health. Globalization is not a neutral process and has been going on for many centuries taking on different iterations during each phase and cycle.

In the past three decades many research and advocacy efforts have targeted neoliberalism for its direct contribution to the widening gap in health inequities that are tied to other aspects of globalization such as racialization, patriarchy, (neo) colonialism, and poverty. Neoliberalism as an ideology yields government actions such as the dismantling of state-provided welfare; the relegation of many social services such as health care onto individuals, families, and communities; and a shift to privileging flexible labour in the market which influences many health professionals.^29^ Feminist and/or decolonial scholars have critiqued neoliberal approaches to citizenship rights and policy and the gendered racialization of precarious, casual, insecure, and unpaid work.^30,31^ Additionally, internationally and nationally recognized health and public policy researchers continue to report on the non-neutral, harmful, violent, re-colonizing, and unjust effects of neoliberal globalization for racialized and Indigenous women’s health and the health of their families in the Global North and South.^32,33^ This is clearly not a new issue, rather a contemporary formation and component of a longer history of modernity built on gendered, capitalist and colonial expansion. The beginnings of the neoliberal movement can be traced back to the end of the Cold War when the United States took the lead in global development efforts through the World Bank and International Monetary Fund using a capitalist, neoliberal framework that privileged predominantly white populations in North America and Europe.^29^ For instance, Southern countries were given/offered/coerced into Structural Adjustment Programs “designed to orient local economies away from production intended to satisfy the needs of local people and towards producing goods for export.”^34^ Twenty-five years later, the neoliberal globalization process has drastically increased racialized and gendered socio-economic differences, while being extremely counterproductive to promises of environmental reparation and protection.^35-37^ Mainstream political and economic ideologies have important implications for Canadian health professional education. As a subset of our health system, which is strongly tied to the Canadian political economy, medical schools and researchers working to improve medical education practices are subject to influence by global ideologies such as neoliberalism. While there are different ideas about their consequences, new programs such as the increased targeting of medical schools by pharmaceutical companies,^38^ establishment of international partnerships to fill vacant medical school seats,^39^ and the widening gap between the salaries of family doctors and specialists^40^ are all examples of neoliberal ideology in medical education. This leaves policy makers, educators, and learners in the paradoxical position of “resolving intractable conflicts between the priorities implied by a focus on health equity and the traditional preoccupations of foreign policy (and the economic interests of national and global elites).”^41^

Internationalization, on the other hand, is a discourse that has been circulating in the research and practice of institutions such as post-secondary education. The Association of Canadian Deans of Education (ACDE) describes the critical issues that institutions of post-secondary education are facing nationally in the *2014 Accord on the Internationalization of Education*.^42^ Overall, internationalization refers to increasing mobility of students, faculty, and staff amongst and between countries; growing number of teaching and research partnerships outside of Canada; and updates to curriculum to equip students to “address complex local and global challenges in socially accountable ways.”^42^ In Canada, 89% of universities state that the pace of internationalization has accelerated in the last three years.^43^ As with internationally governed policies on globalization, the general focus of internationalization in Canadian post-secondary education campuses has been to prepare students for the job market, contribute to the national gross domestic product, while simultaneously promoting global mobility of students, faculty and staff to further improve local economic advantage in the “global knowledge economy.”^44^ In education, Freire describes this as the “banking system” of education where dominant ideologies of material accumulation, western middle-class lifestyles, and entrepreneurship are promoted as central tenets of a happy, healthy world.^45^

One major aspect of internationalization has been the recruitment of international students through “a commitment to ‘brand’ Canada to maximum effect” to enhance Canada’s competitive advantage against other Northern countries.^46^ While the numbers of international students has been rising at a rapid pace, I argue that other aspects of internationalization such as diversification and retention of racialized faculty members (currently only 0.5-5 percent of faculty across Canada are members of racialized groups)^47^ and the inclusion of non-Euro-American-centric curriculum (such as Southern theorists) are not seen as immediate priorities. Under this rubric, the idea of “global” must be problematized as something that does not simply mean the transcendence of nations, but rather signifies a rhetoric of universal humanism based on a hierarchy of geopolitical location, Northern beliefs, and privileging economic benefits over social ones. Often when “global” is deployed, institutions of post-secondary education do not take into consideration colonial histories that have shaped our current, complex world systems. The use and promotion of mainstream “global” projects disguise “the politics and power structures that are tied to the interests of and allegiances to the nation-state.”^48^ Through a neoliberal framework of internationalization, the universality of Euro-American Northern knowledge continues to be asserted across the globe, colonial relations are reproduced through “helping” narratives created through initiatives such as international service-learning, mass produced English as a second language programs, and other international “development” work.^49^ While other articulations of this process exist, in general institutions of post-secondary education are under increasing pressure to transform themselves to compete internationally for enrolments and recognition of excellence in teaching and research.

As stated above, Canada’s specific geographical and socio-political context informs our contemporary position as a comparatively diverse country in the Global North. Linguistically, Canada’s cultural diversity (GI) score is 0.692 in contrast to 0.278 in the US, 0.093 in Australia, and 0.090 in the UK.^50^ In medical education, with the rise in international medical graduates (IMGs) in Canada, increasing diversity in local students, faculty, and staff, and the promotion of affirmative action policies to recruit Black/African, Indigenous (First Nations, Inuit, and Métis) and other racially marginalized students, it is important to consider how a pluralistic approach may be taken to sharing and thinking about medical knowledge. As a leading thinker in comparative post-secondary education, Knight suggests that internationalization requires integration of “an international, intercultural or global dimension into the purpose, function or delivery of post-secondary education.”^51^ In Canada, the imperative to introduce an international lens to medical school policy, pedagogy, and practice is closely aligned with calls for re-prioritizing health advocacy in medical education.^52^ A shift towards a decolonial theoretical perspective to internationalizing curriculum and teaching would necessarily require students to be equipped to respond and promote the needs and self-determined wellness plans of different communities and individuals.

There are no ideal models for politicians, economists, and people to approach globalization, nor post-secondary institutions to approach internationalization. However, in the case of health professions education, research suggests that there is a great need to explicitly address the continual reproduction of North-South power relations. The reproduction of inequitable power relations is caused by using ahistorical, hegemonic, and paternalistic approaches to health professional education. A number of researchers on medical and other post-secondary education in Canada have written about different ethical approaches to global engagements, which go beyond the scope of this paper.^53^ Using my own practice experience working in global health education, the methods of critical literature review, current practice examples from publically available sources on the web, and theoretical advancements in my field of social justice research, the following sections of the paper explore pressing ethical challenges using a decolonial theoretical perspective with implications for all members of health professional education institutions, but particularly for policy makers writing internationalization policies. Initially a search on internationalization in medical education was conducted on Google Scholar, PubMed, and CINAHL, which yielded few results. I pursued papers from the reference lists of these few sources, while also supplementing with the database of sources on this topic I have been collecting since 2011. To begin, I formulate a set of questions grounded on the decolonial orientation described in the previous section. These questions touch upon ethical issues related to the expansion of our frames of reference beyond neoliberal, gendered, and colonial ways of thinking, and to the education of a growing number of health professionals in our diverse country and globe.

### Ethical questions for internationalization policy in medical education

#### Question 1: How can the internationalization of medical curriculum move beyond hegemonic perspectives?

Conceiving medical education through a decolonial and international lens necessitates revisiting the construction of western, Cartesian science as the foundation of our medical programs.^54,55^ Critical scholars also refer to this as a post-positivist approach to education in the natural sciences – an approach that views health through a physical cause and effect relationship, and situates prediction and technical control as the best-practice methodology. While Canadian medical schools have moved to a problem-based learning model and include curriculum on the social determinants of health, there is a dearth of literature and practice that engages with the philosophy of medical knowledge production. From a macroscopic perspective, the strategies used in health professional education are closely connected to our “culture’s guiding vision and narrative of itself.”^45^ In the context of neoliberal globalization discussed above, the recent mainstream Canadian narrative for the past decade has been a return towards a culture of post-positivism that continues to mask the oppressions inherent in a one-dimensional, Cartesian worldview.^56,57^ This socialization and way of thinking links medical education to the broader forces of globalization. Indeed, moving towards decolonial, ethical practices in Canadian medical education entails deeper consideration of what it means for medicine to be an upper-class profession, what it means for students raised in Canada and abroad to assert their position as keepers of medical knowledge, what political economic structures shape the epistemology of dominant medical practices, and what it means for Canada to be a settler-colonial, hegemonic power in the Global North with a history of erasing Indigenous communities worldviews.

Internationalization of medical education curriculum through a decolonial theoretical perspective also requires a connection between locally under-represented, under-served communities and those in the Global South. This can take the form of integrating research critical theories of racialization advanced by scholars who study representation, identity, and racializing structures.^58-60^ Additionally, the internationalization process can harness the knowledge of IMGs who are participating in licencing examinations, clinical education, and as residents. Meaningful collaboration and consultation between Northern and Southern partners on curriculum development requires mindful consideration of the problematic dynamics of the “native informant” that foreclose the possibility of an ethical practice.^61^ As postcolonial scholar Gayatri Chakravorty Spivak observes, the native informant becomes the medium through whom countries of the South can be monitored and disciplined into re-affirming “the social Darwinism implicit in ‘development’ [discourses].”^62^ In other words, IMGs cannot be seen as strictly capital resources or repositories of exotic knowledges for the North. This can easily fall into reproducing and reaffirming the dominant culture’s message and actions. Instead, through a decolonial perspective, IMGs can be considered partners in transnational knowledge production and equal contributors to the health system.

Finally, moving beyond hegemonic perspectives in the internationalization of medical education require meaningful involvement with Indigenous peoples and their ways of healing. An important Canadian example of a counter-hegemonic way of incorporating a pluralist perspective is San’yas Indigenous Cultural Safety (ICS) Training, an educational initiative developed by the Provincial Health Services Authority in British Columbia in response to the Transformative Change Accord First Nations Health Plan signed in 2005.^63^ ICS training is available through a facilitated, online module designed to increase knowledge, enhance self-awareness, and strengthen the skills of those who work both directly and indirectly with Indigenous people. Participants learn about aspects of colonial healing history including Residential Schools and “Indian Hospitals.” Additionally, they are challenged to be reflexive about their own culture, subliminal stereotyping, and the harmful consequences of discrimination. While this example focuses on continuing education of practicing health professionals, it illustrates one way in which multiple worldviews can be incorporated into the broader scope of medical education policy, education, and practice. Addressing the “structural violence” faced by Indigenous peoples (such as disproportionate risk for mental illness, substance use, and sexualized violence) acts as a vanguard for similar historical and contemporary colonial relations such as the increasing fear of and racialized discrimination of Muslims in Canada and elsewhere in the Global North, the erasure of histories of racism towards South Asian and Asian communities, and heightened advocacy and awareness of anti-Black racism in Canada and the United States. These are all issues to consider when preparing learners for practice in an increasingly international field.^64,65^

These critiques in no way discount 150 years of Canadian medicine based on western Cartesian science, but rather display the imperative of exposing medical students to the multiple epistemologies that exist in the world. These will be ways of knowing, healing, and health that they will encounter when working with diverse clients and colleagues in their medical practice. Moreover, a turn towards Southern and Indigenous Canadian epistemologies is necessary to create a healthier relationship with the land and earth’s resources. In fact, the processes of internationalizing curriculum using a pluralist approach allows for an opportunity for developers to be reflexive in thinking about how medical educators can champion the ethics of social accountability, health equity, and anti-oppression. Pluralism differs from a multicultural approach, which underpins current discourses in medical education on cultural competency. Critical anti-racist scholars describe multiculturalism as a decontextualized, depoliticized, and additive approach to thinking about the inclusion of Indigenous and other racialized peoples in Canada.^66^ On the other hand, a pluralistic perspective requires the practice of unlearning and “disenchantment” with a singular future of globalization, education, health care, and society more broadly.^67,68^ It can also encourage innovation by fostering a more dynamic, progressive approach to medical education. A decolonial approach in medical education more broadly can attend to the call for a renewed focus on grounded research and practice in curriculum studies and policy.^69^

#### Ethical question 2: How can we make visible and address repeated patterns of colonial and paternalistic relationships in North-South International Medical Electives?

International electives and programs that target students and youth are one medium through which health professionals engage with global discourses on health systems. In fact, Canadian medical students are increasingly participating in International Medical Electives (IMEs). One of three Canadian medical school graduates have completed an IME during their degrees.^70^ In a review of schools across Canada, I find that the majority have well-established Global Health Offices (GHO) whose main function is to balance this high demand with the ethics of practicing skills and sharing knowledge in another country. GHOs across Canada address the social, political, and economic context of IMEs in different ways. During my experience working at a progressive GHO, important ethical questions came up in many steps of IME planning including pre-departure training, establishing the length and location of placements, and the clinical guidelines during field placement. GHOs are essential stakeholders for writers of internationalization policy to consider as a target audience. From a decolonizing theoretical perspective, the demand for IMEs has obvious implications for internationalization policy and the overall reforms to medical schools through globalization.

In educational studies, critical decolonial theorists describe the “benevolent” tone of Northern global citizenship education. Jefferess very bluntly, but accurately, states that international placements can easily fall into the trap of reinforcing neoliberal, neocolonial, and patriarchal relationships.^71^ For example, attitudes of salvationism can reinforce internalized colonial thinking for both Northern and Southern students, health professionals, and communities. Additionally, the short-term, changing rotations of students, money, and supplies can create a cycle of dependence on foreign medical schools for clinics and hospitals in the Global South. By not acknowledging root causes of a punishingly unfair and uneven distribution of wealth and the continued disenfranchisement of racialized people, especially women all over the globe, Northern medical school’s involvement will tend to reinforce power relations rather than disrupt them. This is analogous to treating the symptoms rather than the causes of illness and unintentionally making the situation worse.

While the personal transformations that often occur during IMEs are real and important,^72^ medical schools become complicit in reproducing inequitable relationships by selling IMEs as a branded experience in “low-income settings” to medical students in the Global North. Moreover, students on IMEs can come across practice situations for which they do not feel adequately prepared or are unsure on how to ethically react largely centering around embedded Euro-American-centric beliefs and material wealth, which questions the necessity for them to be there in the first place.^73^ By thinking of IMEs through a supply and demand perspective, medical schools run the risk of erasing awareness of the ongoing violences of colonialism and causing potential harm to students, clients, and communities, not to mention losing a powerful learning opportunity. This approach can essentialize partnerships between countries to an ahistorical financial transaction. Participation in IMEs can actually do more harm than good.

Despite the mental, emotional, physical, and spiritual difficulties of admitting one’s own complicity in a global system of oppression, the reality of the situation is that this division cuts across and strengthens racialized lines. Postcolonial scholars have documented the centuries’ long history of orientalism, in which the “other” is regarded as exotic, primitive, and in need of help.^72^ From an intersectional, gender-based perspective, this backdrop has rendered countries in the Global South as perpetually “under-developed,” with their racialized populations (in particular women and children) seen as “always-lacking” in contrast to the predominantly white populations of the Global North. A very concerning consequence of this helping narrative overlaid with neoliberal globalization is the slow *erasure* of non-Northern knowledge of healing and medicine. Southern populations are indeed under a constant state of “epistemic violence” as a result of globalization.^74^ Andreotti writes, “the modern/colonial global imaginary consecrates its ‘shine’ (of seamless progress, heroic human agency and evolution as wealth accumulation) while denying its necessary shadow (of violent dispossession, destitution, extraction and genocide).”^75^

In short, Canadian medical schools and those of us working and learning within these institutions should deeply consider our own ancestral and political positions when re-thinking policy and practice. Critical self-reflection, self-awareness and establishing partnerships using the tenets of decolonizing practices means engaging with our own frames of reference (such as our worldviews and the social relations of ethnicity, nationality, gender, sex, class, sexual orientation, ability, etc.) as much as with university partners and communities in the Global South. Collectively we must acknowledge that financial and material poverty across the globe is very closely tied to the interdependent histories of gendered capitalism and colonialism which underpin our contemporary position in a rapidly globalizing world. It also means making connections between the advocacy of racialized groups in Canada and other Northern countries and co-operations in Asia, Africa, Latin America, and the Middle East. For example, members of the medical education community can make links between calls for Indigenous self-determination, acknowledging the environmental costs of IMEs, and considering the numbers of international treaties on which Canada is a signatory.

Through my work and study at several institutions of post-secondary education across the country, I have come across a number of researchers, teachers, and advocates playing a formative role in decolonizing theory and practice. For example, nationally we have the Researchers and Academics of Colour for Equity/Equality (RACE) Network. Regional examples include: the South House Sexual and Gender Resource Centre (Dalhousie University), the Centre for Race, Autobiography, Gender, and Age and Critical Racial and Anti-Colonial Studies Research Network (University of British Columbia), the Critical Research in Health and Health Inequities Research Unit (University of British Columbia), and the Centre for Integrative Anti-Racism Studies (University of Toronto). Understanding and applying tenets of decolonial theoretical perspectives are not easy, linear processes. As I have stated above, the intersections of racialization and global wealth are complicated, messy, and require guidance from and discussion with those at the front lines of social justice work. The expert groups across Canada listed above organize a number of academic conferences and workshops that can be excellent resources for the medical education community.

#### Ethical Question 3: How can we re-examine the question of International Medical Graduate mobility within an uneven context of North-South relations?

Nationally, almost one quarter of all active physicians completed their degree in another country. The numbers of International Medical Graduates (IMGs) in Canada continue to rise as immigration, the demand for rural physicians, and competition in undergraduate and residency admissions increases. It is no secret that MD admissions have become more competitive. Not only are we receiving high number of IMGs from diverse backgrounds, but many more Canadians are going to medical schools oversees with the aim of returning and applying to the Canadian Resident Matching Service (CaRMS).^76^ A province specific study of IMGs from what the World Bank defines as low-income or middle-income countries shows that only 55 percent of these IMGs in Ontario are working as physicians.^77^ The authors describe the underutilization of immigrant skills as a form of “brain drain” that has significant consequences for countries that have low densities of physicians. Misinformation about finding employment in medicine in the North wrenches away health human resources from Southern countries, incurs major financial cost to IMGs and their families, and then underutilizes the medical talent in the new place they have landed. Everyone loses. Additionally, a lack of adequate orientation to the Canadian health care and medical education system for active and non-active IMGs maintains social inequities between Canadian-trained graduates and their (often racialized) counterparts.^78^

While the roots of this problem perhaps lie in the marketing strategy of Canadian immigration, it is also the responsibility of medical institutions corresponding with IMGs to consider their position in the brain drain neo-colonial, enterprise. The top three reasons IMGs cite for moving to Canada are: 1) socioeconomic or political situations in their home countries; 2) better education for children; and 3) concerns about where to raise children. Implicit in these reasons is the understanding that Southern countries are *lacking* in something that can only be acquired by moving to the Global North. While I am not arguing for increased barriers to mobility for those in the Global South, policy writers should consider how this life-style hierarchy is related to colonial histories and a neoliberal approach to education. In summary, when situating the inclusion and exclusion of IMGs in Canada, the medical education community must consider how our privilege in the North has a direct effect on the valuation of medical knowledge and education from Southern countries. Moreover, we must always be aware of the racialized, gendered, and classed ways in which global politics of health human resources operate. Indeed, policy and practice responses should consider the ongoing processes of gendered colonialism and neoliberalism that narrate the push-pull factors of the IMG experience in Canada.

### Conclusion

The paper aims to engage readers with the ethical imperatives and concerns that are necessary considerations for the explicit uptake of internationalization issues in the pedagogy, curriculum, and policy of medical education institutions. As I demonstrate in the sections above, the ultimate paradox for health professional education institutions is the incongruity between: 1) the ethical responsibility for national and global health, wellness, and equity, and 2) the dominant narrative of neoliberal globalization that continues to be a major contributor to health inequities between and within the Global North and South. Through an extensive literature review and through my own professional experience in medical education, I find three important discussions in which this incongruity must be considered. Firstly, when conducting the ongoing process of curriculum review and design, members of the medical education community should consider which epistemological and theoretical traditions and which people are represented in their deliberations. Post-secondary education and critical health literature indicate that the future of medical education requires extending the underpinning epistemology far beyond post-positivism and market-thinking to ensure that multiple approaches to wellness are included and taught to health professionals by diverse teachers. Numerous scholars demonstrate that ahistorical, Euro-American-centric, and paternalistic approaches to globalization have resulted in an erasure and de-valuing of non-Northern scientific definitions of health and illness. Secondly, when building programs and preparing students for IMEs policy makers and educators should consider the ongoing disenfranchisement of racialized and Indigenous groups in Canada and globally. While it is an uncomfortable process to work towards decolonizing North-South relations, acknowledging one’s individual, institutional, and societal complicity is a vital step. Finally, policy makers and educators should reflect the members of the communities they represent and consider the position of IMGs in the “international” network. This is particularly salient in the context of policies that continue to uphold the constant expropriation of values and earth’s resources and the resulting burden of debt for Southern countries. In Canada, such policies are shown to reinforce the cycle of poverty for racialized communities.

There are some limitations to this review of literature and reflexivity tool. Firstly, I take a highly critical view on the consequences of colonialism for racialized communities in the Global North and Global South, which may not be the same worldview as some readers of *CMEJ*. This is informed by my positionality as a member of the Indian diaspora in Canada, my research and practice to date, the countless oral stories I have heard from my racialized colleagues, students, and friends, and the written archives of racialized people across the globe. Secondly, scholars who have shaped the field of decolonial theory are necessarily wary of research methods such as systematic literature reviews, which are premised on positivist and post-positivist epistemologies. As such, this paper takes a critical approach to the literature review using scientific methods such as Boolean searching, and decolonial research methods such as mentorship and conversation with experienced scholars of critical theories of racialization. Overall, this paper aims to provide a literature review, though incomplete and changing, on the implications of decolonizing theoretical perspectives and practice for medical education during a time of intense Global North-South globalization. The review uncovers a number of other ethical considerations where further work is needed. For example, the “research” that is often conducted in the Global South by students and professors from the Global North during international electives and fieldwork can be re-examined through a decolonizing perspective. Additionally, more research can be done to update the empirical evidence on the health costs of inequitable globalization on clients, communities, and our health care systems.
